# Pathogenic Connections in Post-COVID Conditions: What Do We Know in the Large Unknown? A Narrative Review

**DOI:** 10.3390/v14081686

**Published:** 2022-07-30

**Authors:** Celina Silvia Stafie, Sorina Mihaela Solomon, Irina-Georgeta Sufaru, Maria Manaila, Ingrid Ioana Stafie, Gabriela Melinte, Bianca Simionescu, Letitia Leustean

**Affiliations:** 1 Department of Preventive Medicine and Interdisciplinarity—Family Medicine Discipline, Faculty of Medicine, Grigore T. Popa University of Medicine and Pharmacy, 16 Universitatii Street, 700115 Iasi, Romania; celina.stafie@umfiasi.ro; 2 Department of Periodontology, Grigore T. Popa University of Medicine and Pharmacy, 16 Universitatii Street, 700111 Iasi, Romania; 3 Endocrinology Residency Program, Sf. Spiridon Clinical Emergency Hospital, Independentei, 1, 700111 Iasi, Romania; maria.manaila@email.umfiasi.ro (M.M.); stafie.ingrid-ioana@email.umfiasi.ro (I.I.S.); gabriela-melinte@email.umfiasi.ro (G.M.); 4 Pediatric Clinic No. 2, Mother and Child Department, Iuliu Hatieganu University of Medicine and Pharmacy, 8 Victor Babes, 400347 Cluj-Napoca, Romania; bianca.simionescu@umfcluj.ro; 5 Department of Endocrinology, Faculty of Medicine, Grigore T. Popa University of Medicine and Pharmacy, 16 Universitatii Street, 700115 Iasi, Romania; letitia.leustean@umfiasi.ro

**Keywords:** adult multisystem inflammatory syndrome, children multisystem inflammatory syndrome, comorbidities, endocrine disorders, hyperinflammation, long-COVID syndrome, post-COVID conditions

## Abstract

The coronavirus 2019 (COVID-19) disease has long-term effects, known as post-COVID conditions (PCC) or long-COVID. Post-COVID-19 syndrome is defined by signs and symptoms that occur during or after severe acute respiratory syndrome coronavirus 2 (SARS-CoV-2) infection which persist for more than 12 weeks and cannot be supported by an alternative diagnosis. The cardiovascular damage caused by COVID-19 in the severe forms of the disease is induced by severe systemic inflammation, considered to be one of the causes of myocardial lesions, with increased levels of circulating cytokines and toxic response mediators. We have focused on conditions that can induce long-COVID-19, or multisystem inflammatory syndrome in adults or children (MIS-C/MIS-A), with an emphasis on endocrinological and metabolic disorders. Although described less frequently in children than in adults, long-COVID syndrome should not be confused with MIS-C, which is an acute condition characterized by multisystem involvement and paraclinical evidence of inflammation in a pediatric patient who tested positive for SARS-CoV-2. At the same time, we mention that the MIS-A symptoms remit within a few weeks, while the duration of long-COVID is measured in months. Long-COVID syndrome, along with its complications, MIS-A and MIS-C, represents an important challenge in the medical community. Underlying comorbidities can expose both COVID-19 adult and pediatric patients to a higher risk of negative outcomes not only during, but in the aftermath of the SARS-CoV-2 infection as well.

## 1. Introduction

The coronavirus 2019 (COVID-19) disease pandemic made it clear to the world that better preparedness for future pandemics is paramount. First identified in December 2019 in Wuhan, China, the new severe acute respiratory syndrome coronavirus 2 (SARS-CoV-2) infection subsequently spread rapidly in 189 countries within 11 months, leading to a global pandemic, with over 55 million confirmed cases and more than 1 million deaths. Initial reports of the disease highlighted the predominant involvement of adults, with pediatric cases accounting for only 2.1–7.8% of all infected patients [1,2]. However, the actual incidence among children remains uncertain, as most cases develop asymptomatically or through a mild form of the disease [1]. If we could have anticipated the evolution from viral infection to multisystemic autoinflammation, perhaps we could have fought better and wiser against the mortality and long-term morbidity of COVID-19 in the past three years. Systemic inflammation is the key pathophysiology of this infection, especially in moderate and severe cases, with a pro-inflammatory response of the host being responsible for the cytokine surge [3].

COVID-19 has long-term effects, known as post-COVID conditions (PCC) or long-COVID. Patients with long-COVID present with a functional and structural syndrome of several systems, mainly involving general symptoms, tiredness or fatigue that interferes with daily life, symptoms that worsen after physical or mental effort (also known as “post-exertional malaise”), or fever [4]. There can be also respiratory and heart symptoms, such as breathing difficulties or shortness of breath, cough, chest pain, fast-beating or pounding heart (also known as heart palpitations), or worse, neurological symptoms: thinking or concentration difficulties (sometimes referred to as “brain fog”), headache, sleep problems, dizziness when you stand up (lightheadedness), depression, or anxiety.

Globally conducted studies reported different incidence rates for long-COVID syndrome, but also with different monitoring periods of patients after acute infection—76% of people at 6 months post-infection [5] and 32.6% or 87% at 60 days [6,7]. Unfortunately, these data cannot be fully corroborated; however, it was suggested that a substantial proportion of patients with SARS-CoV-2 infection will develop long-COVID syndrome [8].

Risk factors for the development of a severe or critical form of COVID-19 include old age, male gender, and black race as well as pre-existing comorbidities (obesity, cardiovascular disease, and lung disease). At the same time, the people most likely to develop long-COVID-19 syndrome are those who have had a severe or critical form of SARS-CoV-2 infection [9,10].

Over the course of the COVID-19 pandemic, it has become clear that the clinical features, epidemiology, and outcomes of COVID-19 are different in children, compared to adults. A post-infectious hyperinflammatory phase termed multisystem inflammatory syndrome in children (MIS-C) was first reported in the pediatric population in April 2020 [11,12]. The new disease is called “the multisystem inflammatory syndrome” (MIS), due to its physiopathology, and can be fatal if treatment is not started early [13].

In the present review, we shall focus on conditions that can induce long-COVID-19, or even MIS-C/MIS-A, with an emphasis on endocrinological and metabolic disorders. We also propose an evaluation of potential immunologic, hematologic, and cardiovascular complications related to long-COVID.

## 2. Materials and Methods

Pubmed, Science Direct, and Medscape databases were accessed and the following keywords were used: MIS-A and long COVID; long COVID consequences; the relationship between long COVID and MIS-A; the relationship between post COVID and MIS-A; multisystem inflammatory syndrome; long-COVID; chronic COVID; post-COVID; post-COVID-19 syndrome, MIS-C, and the relationship between long COVID and MIS-C. A total of 5460 results were identified, of which, the most important 101 studies between 2019–2022 were reviewed.

Regarding long-COVID syndrome, we cannot say that there is an association between it and MIS, a conclusion supported by the review of 26 studies in the literature, for which we used keywords “long COVID”, “MIS-A”, “MIS-C”, “post-acute sequelae of SARS-CoV-2 infection”, and “post COVID-19”.

## 3. Epidemiology and Pathogenic Factors in MIS

Given the abundance of case definitions and disease definitions in the last two years, confusion in terms linked to COVID-19 is common. Most patients have a complete recovery within approximately 3–4 weeks of SARS-CoV-2 infection, but some of them have experienced persistent symptoms known as long-COVID [14,15]. Currently, there is no consensus on the definition of post-COVID syndrome, so several terms have been used in the literature: long-COVID, long-term COVID, and post-acute sequelae of SARS-CoV-2 infection [16].

COVID-19 long syndrome is a complex of symptoms that appear or persist after acute viral infection, in the absence of other possible diagnoses, over 2 weeks for mild illness, over 4 weeks for moderate-severe disease, and over 6 weeks for critically ill patients [17,18]. It has been hypothesized that a severe form of viral infection is a risk factor for long-term COVID as it causes an exaggerated immune response, and, consequently, multiorgan damage, and the therapeutic approach involves multiple treatment regimens, with possible iatrogenic damage [18].

The World Health Organization estimates that approximately 25% of adult patients infected with the SARS-CoV-2 may develop long-term manifestations. An Italian study suggests the presence of at least 2 post-acute symptoms in 32% of patients and at least 3 symptoms in 55%, while Chinese literature identifies long-COVID syndrome in 76% of individuals 6 months after illness [17].

To date, available data related to long-COVID in children are rare, with 5 pediatric cases with a mean age of 12 years in Sweden, respectively, as reported in an Italian study, whose symptoms persisted 6 months after acute viral infection [14,17].

A study in the literature contrasted a 4 times higher prevalence of long-COVID syndrome in symptomatic children during acute disease (46.5%) compared to asymptomatic ones (11.5%). It appears that symptomatic COVID-19 infection predisposes one to a 6-fold higher risk of developing at least one manifestation of long-term COVID syndrome, particularly respiratory, neurological, or psychological impairment. Furthermore, the incidence of long-COVID was higher in children with cardiac, pneumatological, or neurological comorbidities than in those without significant personal pathological history [17].

The first pediatric cases of MIS were reported in the UK, specifically in a group of eight previously healthy children who had hyperinflammatory shock syndrome temporarily associated with COVID-19 infection [19]. Subsequently, countries such as Italy, Spain, France, and Switzerland were affected, and the general manifestations were similar to already known entities, such as Kawasaki disease, toxic shock syndrome, and macrophage activation syndrome [2,20]. To date, we were not able to find reported cases of MIS-C in China or Japan in the English literature [20]. On the other hand, the real incidence of MIS-A is not known, being rarely reported, but has mostly been found in males (73.4%), with a mean age of 31 ± 10 years, mostly Asian (25.4%), Caucasian (23.6%), and Hispanic (21.8%) descent, with onset about 2–5 weeks after initial SARS-CoV-2 infection [21,22].

Feldstein et al. compared the clinical characteristics and outcomes of children and adolescents with MIS-C versus those with severe coronavirus 2019 (COVID-19) disease, in 1116 patients under 21 years old and hospitalized between March 15 and October 31, 2020, at 66 US hospitals in 31 states [23]. The authors concluded that patients with MIS-C were more likely to be 6 to 12 years old and are more likely to have cardiorespiratory involvement {56.0% vs. 8.8%; RD, 47.2% (95% CI, 42.4–52.0%); aRR, 2.99 (95% CI, 2.55–3.50)} vs. respiratory involvement or cardiovascular involvement without respiratory complications {10.6% vs. 2.9%; RD, 7.7% (95% CI, 4.7–10.6%); aRR, 2.49 (95% CI, 2.05–3.02)} vs. respiratory involvement. Moreover, important predictive laboratory data showed that patients with MIS-C had a higher neutrophil to lymphocyte ratio (median, 6.4 vs. 2.7, *p* < 0.001), higher C-reactive protein level (median, 152 mg/L vs. 33 mg/L; *p* < 0.001), and lower platelet count (<150 × 103 cells/μL) [23].

**Figure 1:**
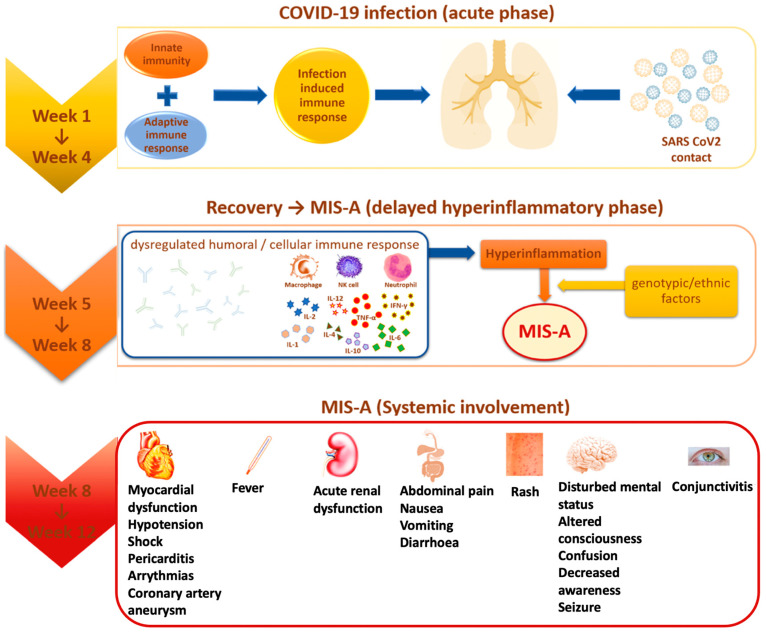
MIS-A evolution and systemic involvement.

Multisystem inflammatory syndrome (MIS) is a rare, but serious condition associated with COVID-19, in which different body parts become inflamed, including the heart, lungs, kidneys, brain, skin, eyes, or gastrointestinal organs (Figure 1). MIS can affect children (MIS-C) and adults (MIS-A). MIS-C case definition includes people who are younger than 21 years old, and the MIS-A case definition includes people who are 21 years and older [13]. The exact prevalence of Multisystem Inflammatory Syndrome in Adults (MIS-A) is largely unknown [22]. With all that we know from April 2020 until May 2022, MIS-A and MIS-C can be treated if an early diagnosis is performed and therapeutic intervention is immediately set. Both MIS-C and MIS-A have 81% cardiac symptoms, the rest is divided into gastrointestinal (73.4%), and dermic and musculoskeletal symptoms (51.9%) [22].

In the second half of April 2020, a new syndrome was highlighted for the first time in the pediatric population aged 8 to 15 years, called “multisystem inflammatory syndrome in children” (MIS-C), manifested by fever, hypotension, severe abdominal pain, and heart dysfunction, present in patients who tested positive for COVID-19 infection [20]. Subsequently, in June 2020, similar symptomatology was identified among adult patients, but although there were several reported cases, it was not until August 2021 that it was confirmed as “multisystem inflammatory syndrome in adults” (MIS-A) through laboratory tests [21,24].

In multisystemic inflammatory syndrome (MIS), SARS-CoV-2 infection is identified by rapid antigen testing, positive RT-PCR, or direct exposure in the last 4 weeks before the onset of symptoms [1,2].

Most of the identified MIS cases showed positive serological tests expressed by the presence of specific antibodies (84% in MIS-C and 85.3% in MIS-A), and to a lesser extent, positive RT-PCR testing (37% in MIS-C and 36.4% in MIS-A), an aspect that suggests the appearance of the multisystemic inflammatory syndrome as a post-infectious consequence and not directly attributed to early acute infection [2,19,22]. This observation is supported by the fact that most cases of MIS occurred after about 2–4 to 6 weeks compared to the peak incidence of COVID-19 in some countries [1,19]. In patients who have not reported previous respiratory symptoms of viral infection, it is difficult to estimate the time interval between infection and the development of MIS-A, so the question arises: is MIS-A a manifestation of acute infection or a post-acute phenomenon [25]?

Significantly higher concentrations of antibodies directed against the spike protein receptor binding domain (RBD) of the new coronavirus were observed in hospitalized patients with MIS-C. RBD is the part that mediates the binding of the virus to its receptor, the angiotensin 2 conversion enzyme, on host cells. The number of anti-RBD antibodies is directly correlated with the ESR value, so an increased serological value is associated with a significant proinflammatory state [26].

Even if SARS-CoV-2 is defined mainly by acute severe respiratory symptoms, MIS-A and MIS-C have predominantly cardiac symptoms; tachycardia was the first symptom on admission for 89.7% of cases [27]. There are predictor markers for MIS-C and the study by Merckx et al. pointed out that age and higher ferritin levels were associated with more severe MIS-C.

Multisystem inflammatory syndrome in children (MIS-C) manifests as immune dysregulation after SARS-CoV-2 infection [28]. The syndrome has no pathognomonic features. Thus, the diagnostic criteria of the Royal College of Paediatrics and Child Health (RCPCH), the Centers for Disease Control and Prevention (CDC), and the World Health Organization (WHO) differ, but they all include fever, evidence of systemic inflammation, and involvement of at least one organ or system [29].

Data obtained from all the studies suggest that inflammatory markers were elevated in a majority of cases, with reported leukocytosis and elevated CRP, and percentages from 81.8% to 98.2% for CRP [30]. Lymphopenia was observed in 67.5% of cases. Increased serum ferritin, leukopenia, lymphopenia, and thrombocytopenia are common in MIS-C [31]. The study by Kunal et al. reveals that elevated cardiac troponin was reported in 86%, while elevated brain natriuretic peptide (BNP) and NT-pro BNP were observed in 94.1% and 93.3% of patients, respectively [22].

The Royal College of Pediatrics and Child Health describes this syndrome as a “pediatric inflammatory multisystemic syndrome temporarily associated with COVID-19” (PIMS-TS) [32]. The risk of developing PIMS-TS is 0.14% after confirmed SARS-CoV-2 infection, and mortality is between 1.7–2.2%. The maximum age is between 7 and 10 years, but infants and adults can be affected. Most patients with PIMS-TS do not have comorbidities. Male children and adolescents appear to be affected more frequently, and obesity is mentioned as a risk factor. Differential diagnoses include Kawasaki syndrome, severe infections, toxic shock syndrome, appendicitis, myocarditis, macrophage activation syndrome, and Still’s disease [33]. PIMS-TS occurs approximately 2 to 6 weeks after an often asymptomatic SARS-CoV-2 infection. An excessive reaction of the immune system could be a key factor, but different from the pathophysiology of COVID-19 and Kawasaki syndrome. One possible explanation is the “antibody-dependent amplification” hypothesis. These antibodies bind to the spike protein of SARS-CoV-2 and lead to the increased uptake of the antibody-antigen complex into the target cell, implicitly linked to increased virus replication [34]. Another hypothetical hypothesis relates to a storm of cytokines through a delayed response to interferon. Coronaviruses are able to induce the production of autoantibodies that block interferon type I and interferon III, leading to a delayed response to interferon and, thus, delaying control of the virus, followed by excessive inflammation and the storm of cytokines. Inflammatory cytokines have been increased, especially interleukin (IL) -1β, IL-6, IL-10, IL-13 and tumor necrosis factor (TNF) -α [12].

The study conducted in 2021 by Zhang refers to the similarities and differences between MIS-C associated with COVID-19 and Kawasaki disease (KD); cardiac manifestations are more common than KD, including myocarditis with cardiac dysfunction and coronary artery dilation or aneurysms [31]. The severe cases present cardiogenic shock which requires fluid resuscitation, muscular support, and even mechanical ventilation and extracorporeal membrane oxygenation (ECMO), while KD rarely requires such treatment. The overall presentation and treatment of MIS-C appear to overlap with KD, however, there are still great differences between the syndromes, and it is controversial to say whether MIS-C is a new entity or is a “severe type” of KD [31].

Recent pediatric studies have shed light on the clinical epidemiology of coronavirus 2 severe acute respiratory syndrome (SARS-CoV-2) in children, identifying a high prevalence of asymptomatic and mild infections, with severe COVID-19 infrequently reported. The diagnostic criteria for MIS-C are presented in Table 1.

There are underlying medical conditions, and comorbidities, that have to be considered when deciding to hospitalize a child with COVID-19. The assessment of whether specific comorbidities confer an increased risk for severe disease is challenged by inconsistent reporting of comorbidities, lack of relevant control groups in many published reports, and lack of studied clinical outcomes. In contrast to COVID-19, significant comorbidities are relatively infrequent among those with MIS-C [23,35,36].

The goals of PIMS-TS treatment are achieved by: (A) the stabilization of patients in critical, life-threatening conditions by supportive treatment; (B) the prevention of long-term sequelae (coronary artery aneurysms, myocardial fibrosis) by immunomodulation, associated with (C) anticoagulation medication and (D) antibiotic therapy [13].

The supportive treatment includes:

(a) Volume restoration in patients presenting signs of ventricular dysfunction; fluid boluses of 10 mL/kg should be administered with the reassessment of the signs of shock and fluid overload after each bolus.

(b) Inotropic support may be necessary because most children with PIMS-TS show refractory shock to the administration of liquids. Epinephrine or Norepinephrine is the preferred inotropic-vasoactive medication. The association of Milrinone can be useful in severe cases.

(c) In fulminating forms of the disease, extracorporeal membrane oxygenation (ECMO) is required.

(d) Respiratory support may be necessary to maintain the blood oxygen saturation (SpO2) above 90%.

(e) Renal replacement therapy may be also required.

(f) Plasmapheresis can play a beneficial role in cases of severe cytokine storm, refractory vasoplegia, and cardiogenic or septic shock.

The immunomodulatory treatment is administered in case of suspicion of PIMS-TS with a clinical-paraclinical picture of medium/severe disease, including patients who have no clinical signs of shock but have tachycardia and elevated NTpro-BNP.

The first line of immunomodulatory treatment includes intravenous immunoglobulins associated with Methylprednisolone. Immunoglobulins are recommended intravenously as 2 g/kg over one day or 1 g/kg/day administered on two consecutive days [13]. Intravenous Methylprednisolone is administered at a dose of 1.6–2 mg/kg/day divided into two equal doses every 12 h (maximum 60 mg/day). After 5 days, it can be switched to oral cortisone therapy. It is recommended to continue the cortisone treatment for 2–3 weeks, or even more, depending on the course of the symptomatology, then the dose will be progressively reduced. When the PIMS-TS is unresponsive to intravenous immunoglobulins and glucocorticoids (the fever persists for more than 24–36 h and/or the patient shows significant dysfunction of an organ), pulse therapy with Methylprednisolone is necessary at a recommended dose of 10–30 mg/kg/day in endovenous infusion (maximum 1 g/day) for 1–3 days, followed by oral Prednisone 1–2 mg/kg/day for 7 days, with subsequent dose reduction in an interval of 2–8 weeks, depending on the severity of the symptomatology and their laboratory results [13].

A second dose of immunoglobulins is not recommended for a disease refractory to the initial dose because of the risk of volume overload in a patient with cardiac dysfunction.

A second line of immunomodulatory treatment may be needed for the refractory forms, or if there is a secondary hemophagocytic lymphohistiocytosis (HLH) association [13].

Recent research recommends an immunomodulatory treatment of both Tocilizumab and Baricinitib; both medicines should be taken in combination with glucocorticoids [13].

Therapy with anticoagulants/antiplatelet agents includes aspirin, administered at a low dose (3–5 mg/kg/day, max 81 mg/day) until the platelet count returns to normal and a heart ultrasound performed 4 weeks after diagnosis confirms that the coronary arteries are normal. Aspirin is contraindicated in children with active bleeding or risk of active bleeding, or in case of thrombopenia ≤ 80,000/mm^3^. When the patient has a higher risk of thrombosis (especially depending on the Z-score of the coronary aneurysms), the necessity of subcutaneous Enoxaparin is also discussed [13].

Empirical antibiotic therapy should be given to all patients with sepsis suspicion until the final diagnosis is established. Bacterial superinfection is rare but possible in a child with PIMS-TS [12].

## 4. Comorbidities and Cofactors

Common comorbid medical conditions among the severe COVID-19 patients included obesity (41.8%), respiratory conditions (26.2%), neurologic conditions (18%), and cardiovascular disease (9.8%) [36]. Other comorbidities, defined as the aggregate sum of malignancy, immunocompromising condition, hematologic, renal, gastrointestinal, endocrine, or metabolic conditions, were present in 38.6% of the cases [23].

For adults, comorbidities are wider and very heterogenous, from endocrine conditions to cardiac impairment, or even dental and periodontal conditions which play an important role in MIS development. Wang et al. [37] noted that periodontitis was associated with a significantly higher susceptibility to COVID-19; in addition, periodontal disease was significantly related to the severity of COVID-19 (IVW, OR = 1.025, *p* = 0.039, 95% CI 1.001–1.049; weighted median, OR = 1.030, *p* = 0.027, 95% CI 1.003–1.058) [37]. Additionally, Gupta et al. [38] observed that poor periodontal status correlates with an increased risk of hospitalization and ventilation necessity, developing COVID-19 pneumonia, and dying after SARS-CoV-2, compared to positive COVID-19 patients; severe periodontitis resulted in a 7.45-fold higher risk of requiring assisted ventilation, 36.52-fold higher risk of hospitalization, 14.58-fold risk of death, and a 4.42-fold risk of COVID-19-related pneumonia [38]. Similarly, Marouf et al. [39] found that periodontitis was associated with higher risks of hospitalization, need for assisted ventilation, and death among patients with COVID-19, after adjusting for age, gender, BMI, smoking, and other conditions.

The most frequently proposed pathophysiological mechanism for the link between periodontitis and COVID-19 is based on a series of inflammatory cascades characteristic of both pathologies; thus, the inflammatory components of periodontal disease (TNF-α, IL-1 α, IL-1 β, and IL-6) could be systemically disseminated through the bloodstream, adding an additional burden to the cytokine storm encountered in severe forms of COVID-19 [40,41,42,43].

Another etiopathogenic pathway involves superinfection with aspirated periodontopathogenic bacteria [44]; high concentrations of oral bacteria were determined by sequencing the bronchoalveolar lavage fluid meta-transcriptome from severe COVID-19 patients [45]. In addition, SARS-CoV-2 has been identified in the saliva of positive patients; thus, by aspiration of saliva, the virus can be transported to the lower respiratory tract, increasing the risk of developing more severe forms of the disease [46].

A third potential etiopathogenic explanation is based on the patient’s prolonged exposure to lipopolysaccharides of periodontal pathogens, compounds that can accelerate cellular aging, including the lung epithelial cells; these phenomena can subsequently lead to viral entry and replication, with unfavorable patient evolution [47].

At the same time, periodontal disease shares a significant number of risk factors with COVID-19. Diabetes mellitus [48], obesity [49], smoking [50], chronic kidney disease [51], or atherosclerosis [52], have all been associated with exacerbated inflammatory status in patients with periodontal disease and more severe periodontal tissue damage than in systemically healthy patients. This relationship requires further investigation, as it could positively affect the recognition and early intervention of people at risk of negative end-points.

Thus, we emphasize once again the importance of ensuring a healthy status of the oral cavity, which implies awareness of the patient, but also of medical specialists, as well as the necessity of adequate means of periodontal therapy both at home and in the dental office.

## 5. Immune and Hematological Implications

In terms of biomolecular mechanisms, a number of factors in severe forms of COVID-19 are associated with a specific cytokine profile such as stimulation of interferon production, secretion of interleukin 2 and interleukin 7, granulocyte activation, and TNF production [53]. The systemic inflammatory response associated with SARS-CoV-2 infection may in turn have repercussions on the musculoskeletal system. Numerous studies reveal systemic increases in cytokines and signaling molecules such as CKCL19, IFN-γ, IL-1 β, IL-6, IL-8, IL-17, and TNF-α, secondary to SARS-CoV-2 infection. These molecules have many potential mechanisms by which they can cause musculoskeletal symptoms. IFN-γ, IL-1 β, IL-6, IL-17, and TNF-α affect skeletal muscle by inducing proteolysis of muscle fibers and by decreasing protein synthesis. IL-1B and IL-6 can cause fibrosis by increasing the activity of muscle fibroblasts. IL-1B and TNF-α inhibit the differentiation and proliferation of satellite cells and progenitor cells involved in muscle fiber growth. CXCL10, IL-17, and TNF-α induce osteoclastogenesis and inhibit osteoblast proliferation and differentiation, causing bone fragility. IL-1 β, IL-6, and TNF-α cause chondrolysis manifested by arthralgia and/or osteoarthritis progression. Therefore, the decrease in muscle strength and endurance associated with COVID-19 could be a consequence of the activity of these inflammatory molecules [54]. All these factors cause excessive intravascular inflammation, with changes in angiogenesis and coagulation. At the same time, the association of symptoms with autoimmune pathologies indicates that SARS-CoV-2 can trigger secondary pathologies, associated with a temporary status of immunosuppression and with the presence of the virus [55].

The immunological events associated with SARS-CoV-2 are characterized by the evolution of acquired immunity to the virus, mediated by T and B lymphocytes. Guillain–Barré syndrome has also been associated with COVID-19 [56]. This pathology is characterized by rapid evolution, with the appearance of an inflammatory cascade at the level of peripheral nerves and the destruction of myelin sheaths, being thus associated with polyneuropathy. Guillain–Barré syndrome has been reported in several studies in both pediatric and adult populations, during or after acute SARS-CoV-2 infection, with symptoms ranging from mild to severe respiratory complications. According to the study authors, these symptoms were associated with the physiological stimulation of inflammatory cells, a consequence of SARS-CoV-2 infection [57,58].

Acute COVID-19 infection is associated with hematological complications [59]. Some hematological manifestations usually persist and may worsen in the long-term stage of COVID, such as venous thromboembolism, disseminated intravascular coagulation, thrombocytopenia, autoimmune hemolytic anemia [60], Evans syndrome [61] and hemophagocytic lymphohistiocytosis [62].

Recent post-mortem studies in adults have shown the presence of microthrombi, endothelial inflammation, and platelet activation in viral lung disease, even in the absence of macroscopic embolic events, an observation that supports unsatisfactory clinical results in some individuals, irrespective of the occurrence of post-COVID 19 sequelae [63].

The pathophysiological cascade of SARS-CoV-2 infection involves essential organs and systems in maintaining homeostasis. SARS-CoV-2 causes an excessive inflammatory response which induces the endogenous production of substances that will alter normal vascular homeostasis [55]. Thus, coagulation is directly affected by the release of procoagulant and proinflammatory cytokines, leading to the appearance of disseminated intravascular coagulation and thromboembolic status with secondary damage to tissues, and, in particular, to tissues sensitive to ischemic processes—pulmonary, cardiovascular, and cerebrovascular [64].

## 6. Cardiovascular Complications

Regarding the pathophysiological mechanisms of the cardiovascular damage caused by COVID-19, severe systemic inflammation is considered to be one of the causes of myocardial lesions, with increased levels of circulating cytokines and toxic response mediators—IL-6, TNF, and nitric oxide, which modulate the activity of calcium channels [53]. It is supposed that the action of these mediators generates myocardial depression in cases of excessive inflammation. At the same time, the studies describe direct myocardial viral invasion as an additional mechanism in which the viral particles penetrate the myocardial fibers [23].

The long-term effects of SARS-CoV-2 infection often involve the cardiovascular system. The most common symptoms reported by patients are fatigue, shortness of breath, and chest pain, symptoms which could be explained by persistent myocardial inflammation after acute infection [65]. Another study by Xiong et al. observed the presence of resting tachycardia and palpitations in patients who had experienced acute forms of COVID-19 [66].

Moreover, studies have shown the onset of postural orthostatic tachycardia syndrome which occurs in patients with a history of SARS-CoV-2 infection, secondary to autonomic dysfunction [8].

Two studies investigated groups of young, athletic patients, 2–3 months after acute SARS-CoV-2 infection; the presence of myocarditis was found on cardiac MRI in about half of the study group, although the subjects showed only mild symptoms or were even asymptomatic [65,67].

Another study on 100 patients reported that 78% of them had cardiac changes evidenced by cardiac MRI 2–3 months after the onset of the disease, and 60% had myocardial inflammation, regardless of severity and course of the acute infection [68].

The possibility of cardiovascular lesions in patients with long-COVID syndrome is supported by another study which reported that more than 40% of patients with SARS-CoV-2 infection experienced pericarditis or myocarditis 70 days or more after the infection onset [68,69]. Studies in the literature have shown the involvement of the cardiovascular system in MIS as the main target of the disease (81%) in adult patients, manifested by hypotension, heart dysfunction, cardiogenic shock, myocarditis, anterior chest pain, pericarditis, cardiac arrhythmias, and mitral regurgitation. Echocardiographically, the majority of patients had a reduction in the left ventricular ejection fraction (LVEF ≤ 50%), reversible in 56.1% of cases a few weeks after MIS-A, suggesting that this is part of the systemic inflammatory response rather than a consequence of ischemia or viral myocarditis [22,70].

On the other hand, echocardiographic examinations performed in children with MIS-C objectified cardiac damage in the form of myocarditis, left ventricular dysfunction, and coronary dilation, but gastrointestinal complaints were most frequently reported in this age group [19]. Children with MIS-c who underwent an exploratory laparotomy were found to have mesenteric lymphadenitis which confirms the existence of an active inflammatory reaction in the digestive system. At the same time, in viral infection, enterocytes can be infected by the SARS-CoV-2, and this element supports a possible link between the two pathologies [20].

Paraclinically, both long-term COVID-19 syndrome and MIS are characterized by elevated C-reactive protein, ESR, fibrinogen, ferritin, procalcitonic, leukocytosis or lymphopenia, and increased NT-proBNP, while leukopenia with lymphopenia was the most common change reported in patients with MIS-C [15,22,71]. In addition, a study in the literature showed that the value of D-dimers has an important role in predicting a more severe form of COVID-19 infection and has been increased in patients who have developed long-COVID syndrome [63].

Apart from the data with which the literature has brought us up to date, another angle from which we could look at the two syndromes, long-COVID and MIS, is to turn our attention to the pathologies that can generate cardiac pathology, through their own pathophysiology, with an additive risk for the precipitation of a severe form of SARS-CoV-2 disease. The idea came especially in the context of the appearance of MIS, in 2020, when we found that a disease with a pulmonary debut, in which the main mortality peak was pneumonia and hyper coagulant status, degenerates into life-threatening pathologies which present not the clinical picture of the respiratory system, but heart damage signs and symptoms. We began to explore the endocrine pathology mentioned as the basis for severe forms of SARS-CoV-2, especially since the highest proportion of cardiovascular diseases caused by associated diseases, in particular ischemic heart disease, results from endocrinopathies.

## 7. Endocrine and Metabolic Considerations in PCC

Endocrinologists contributed considerably to the profiling of an endocrine phenotype of PCC, choosing to be in the first line of care for affected patients, in partnership with other physicians such as those in internal medicine and emergency departments. Indeed, as the understanding of COVID-19 has grown, a substantial endocrine and metabolic role has become clearer, with numerous endocrine organs, tissues, and molecules being directly or indirectly influenced or adversely impacting COVID-19 outcomes. In both the general population and patients suffering from various endocrine illnesses, a well-defined endocrine phenotype can help in maintaining health and preventing negative COVID-19 evolution [72].

COVID-19 must exhibit both ACE-2 receptors and the serine protease TMPRSS2 in order to enter cells [73].

Most of the endocrine tissues expressing these receptors are involved during and after recovery from the COVID-19 infection, namely, the hypothalamus, pituitary, thyroid, adrenal, gonad, and pancreatic islets [74]. There has been recent evidence that the endocrine system is vulnerable to both destruction and dysregulation because of COVID-19 [75,76].

Among the hypothesis that could explain the pathophysiology of long-COVID, there are several endocrine dysfunctions leading to the dysregulation of hypothalamus-pituitary-adrenal and hypothalamus-pituitary-thyroid axes, and post-inflammatory hypocortisolism [75]. The contribution of the dysfunction of the endocrine system to long-COVID is still unknown, and future research is needed [76].

### 7.1. COVID-19 and Thyroid Disorders

COVID-19 infection can trigger various thyroid disorders, which can manifest as hyperthyroidism or hypothyroidism. Both the excess and the lack of thyroid hormone can have an important impact on cardiac function. The incidence of thyroid disorders and hypothyroidism secondary to pituitary dysfunction associated with COVID-19 may be linked to the fact that both follicular and pituitary cells present ACE-2 receptors which SARS-CoV-2 can bind to, thus becoming a target for the virus [77,78]. Postmortem studies showed that both follicular and parafollicular cells of the thyroid gland were extensively damaged in patients who died of SARS-CoV-2 [76].

Thyroid dysfunction associated with SARS-CoV-2 infection can also be triggered by the excessive release of pro-inflammatory cytokines associated with the coronavirus in the acute setting. Increased interleukin-6 (IL-6) has been linked to thyrotoxicosis, as shown in a retrospective study where serum IL-6 values were higher in patients with hyperthyroidism compared to those with normal thyroid function [79].

SARS-CoV-2 has been linked to the development of new or recurring autoimmune thyroid disease. The acute inflammatory reaction to SARS-CoV-2 is thought to initiate an immune response that causes Graves’ disease reactivation. Different types of thyroiditis, such as inflammatory or subacute thyroiditis, and autoimmune hyperthyroidism, are included in the differential diagnosis of thyrotoxicosis after COVID-19. Graves’ disease might be caused by an immune cascade generated by the SARS-CoV-2 infection’s hyperinflammatory disease state. Th1 cytokines and IL-6 appear to be involved in the inflammatory response during COVID-19. Graves’ disease is caused by a Th2 autoimmune response; however, elevated levels of IL-6 have been linked to the condition in the past. Stress may also have a role in the pathophysiology of Graves’ disease recurrence after COVID-19 [80].

Graves’ disease is an autoimmune condition where TSH receptor-stimulating antibodies bind to the thyroid tissue, leading to hyperthyroidism. The dysregulated immune system post-COVID-19 infection has been associated with both relapse and onset of Graves’ disease and may even precipitate a thyrotoxic storm in the elderly [81]. A thyroid storm is an exacerbation of thyrotoxicosis, presenting with tachycardia, agitation, altered mental status, gastrointestinal symptoms, and hyperthermia, which can be fatal and require emergency treatment. A thyroid storm can be triggered in people with decompensated thyrotoxicosis by respiratory infections, such as COVID-19, and can increase mortality, secondary to cardiovascular complications [82].

The thyroid hormone in its active cellular form, triiodothyronine (T3), acts both by binding to nuclear receptors in cardiac myocytes, regulating target genes that play an important role in increased contractility and diastolic function, as well by having extranuclear actions, regulating certain sodium, potassium, and calcium channels of the myocardium. This will ultimately lead to increased inotropism and chronotropism and increased cardiac output up to 50–300% higher than in euthyroid subjects [83,84]. T3 also acts on the arterioles of the peripheral circulation by dilating them, thus decreasing the systemic vascular resistance which will result in reduced arterial filling volume. This then leads to the activation of the angiotensin-aldosterone axis, therefore increasing renal sodium reabsorption, with a subsequently higher plasma volume [84,85].

In patients with hyperthyroidism, atrial arrhythmias are more common than ventricular disturbances. Atrial fibrillation has been noted in up to 15% of thyrotoxic patients and even transient thyrotoxicosis in COVID-19 patients can favor arrhythmias which can increase cardiovascular complications [83]. Patients with subclinical hyperthyroidism may exhibit a three times higher risk of developing atrial fibrillation compared to euthyroid patients [86]. Atrial fibrillation can be spontaneously remitted in cases where the thyrotoxicosis is treated, as quoted in the study by Nakazawa et al. where atrial fibrillation spontaneously converted to sinus rhythm in 101 out of the 163 patients once they achieved euthyroid state [87]. Electric or pharmacological cardioversion can be initiated only in patients which have been brought back to a euthyroid state [83].

L-Thyroxine (T4) has been cited to play a role in human platelet activation, which may explain one of the pathways that trigger pathological clotting in overt hyperthyroidism associated with COVID-19 infection [88]. Increased plasmatic concentrations of IL-6, IL-12, IL-18, fibrinogen, VCAM-1, and von Willebrand factor, reported in patients with hyperthyroidism and COVID-19 infection, may also contribute to the hypercoagulability state and increase the risk of thromboembolic events [89].

It should be noted that free thyroid hormones can be increased in individuals who received low-molecular-weight heparin therapy, which is a result of non-esterified fatty acids competing for binding sites on Thyroxine Binding Globulin (TBG). Therefore, free T4 (fT4) and free T3 (fT3) should not be routinely measured in patients taking heparin, and the euthyroid status can be checked by total thyroid hormones and TSH level dosing, when necessary [90].

Heart failure in thyrotoxic patients is associated with high cardiac output and contractility, contrasting the classic characteristics of true heart failure [83]. However, classic heart failure has been described in patients with untreated thyrotoxicosis, chronically exposed to excessive levels of thyroid hormones, who have a history of persistent sinus tachycardia or atrial fibrillation, and present with low cardiac output and decreased myocardial contractility. Early treatment of hyperthyroidism can prevent the onset of congestive heart failure [91].

Underlying ischemic disease or chronic hypertension may also precipitate the heart failure onset in thyrotoxic patients. Individuals with chronic heart failure may present symptoms worsening in the setting of COVID-19-induced subacute thyroiditis [83].

Some authors suggest that thyroid function should be assessed in all COVID-19 patients placed in intensive care units, as they may present with thyrotoxicosis caused by subacute thyroiditis, or even hypothyroidism, which requires levothyroxine replacement [92].

Chronic autoimmune thyroiditis may be the result of immune dysregulation following COVID-19 infection, which may manifest with hypothyroidism. This hypothesis is sustained by a study conducted by Lui et al. where 5.5% of COVID-19 patients who initially had negative anti-thyroperoxidase (anti-TPO) antibodies, tested positive at a median of three months post-infection when their thyroid function was reassessed [93].

In terms of cardiovascular changes, hypothyroidism is associated with bradycardia and low contractility, both resulting in low cardiac output. Systemic vascular resistance will increase and the individuals will present with diastolic hypertension, both changes resulting in higher cardiac afterload. Heart failure is uncommon because hypothyroidism is also associated with a lower need for oxygen supply in the periphery. Hypothyroidism is associated with high levels of cholesterol, which may contribute to increased atherosclerosis. A prolonged QT interval, which can be associated with hypothyroidism, can lead to fatal arrhythmias, such as torsade de pointes [83].

Nonthyroidal illness syndrome, which can be present due to the release of a cytokine cascade, has also been described in patients infected with COVID-19. The typical findings are low plasma T3 and a high level of reverse T3 (rT3); both TSH and plasma T4 may be normal or low [88]. Although this syndrome is associated with higher mortality, it should not be considered overt hypothyroidism and should not be treated with thyroxine. Once the patients recover, their hormone levels returned to baseline [94]. In summary, patients with COVID-19 infection may present an acute phase of various thyroid disorders, including subacute thyroiditis, autoimmune thyroiditis, or nonthyroidal illness syndrome. These thyroid dysfunctions may negatively influence the evolution and prognosis of the acute phase of the disease. Even though long-term studies are lacking, current evidence shows that in most cases thyroid function will return to normal, with no contribution to long-COVID syndrome pathophysiology [76].

### 7.2. COVID-19 and Diabetes

Diabetes is not only one of the most common comorbidities among hospitalized patients with severe COVID-19, but it is also becoming clear that diabetes and COVID-19 may have a bidirectional association. Because pancreatic beta-cells overexpress the ACE-2 receptor, SARS-CoV-2 may harm them, resulting in increased hyperglycemia in individuals with diabetes. Furthermore, new-onset diabetes in previously nondiabetic COVID-19 individuals has been regularly documented, indicating that SARS-CoV-2 may have a diabetogenic effect [72].

Influenza virus infection and replication are considerably increased when pulmonary epithelial cells are exposed to glucose in vitro and raised glucose levels hinder the antiviral immune response [95]. Diabetes is linked to several structural alterations in the lungs in viral illness animal models, including increased vascular permeability and a collapsed alveolar epithelium. Hyperglycemia has been shown to impact pulmonary function, and COVID-19-induced respiratory dysfunction is aggravated in diabetic individuals. In fact, diabetic patients had poorer COVID-19 pneumonia radiologic scores. Glycemic control appears to be linked to outcomes, according to newer research [72]. Hypocalcemia is highly prevalent and predicts hospitalization in patients with COVID-19 [96]. A recent meta-analysis showed that hypocalcemia is correlated with disease severity and mortality in COVID-19 patients, suggesting a relationship between low calcium levels and inflammation [97]. Studies are indicating that patients with COVID-19 and hypocalcemia have an increased inflammatory response, with lower lymphocytes and higher D-dimer levels [97].

Recent evidence shows that persistent hyperglycemia may be found in a significant proportion of patients at 6 months, or even 3 years, following SARS-CoV-2, and direct damage to pancreatic beta-cells structure and function may be one of the incriminated mechanisms for long-COVID syndrome [76].

### 7.3. Hypocalcemia

Because hypocalcemia in COVID-19 can be severe and life-threatening, all patients with postsurgical hypoparathyroidism should be closely monitored and should never stop or reduce their therapy (for example, due to adverse effects) without endocrinologic advice. Furthermore, individuals with moderate hypoparathyroidism who are not on long-term medication, particularly those who are overweight or obese, should be closely monitored in locations where COVID-19 is prevalent and appropriately treated to avoid acute hypocalcemia. Because hypocalcemia may have a deleterious influence on cardiac and neurologic outcomes, calcium testing and monitoring are suggested for all hospitalized patients with SARS-CoV-2 infection [96,97].

### 7.4. Low Levels of Vitamin D

Low levels of vitamin D, corrected for confounding variables, have been identified in a significant number of hospitalized COVID-19 patients with average levels lower than the general population, in several investigations from different geographical locations. Poor vitamin D status has also been linked to severe COVID-19 endocrine comorbidities and has been documented in most studies to predict disease severity, including chest CT stage and mortality risk [98]. Even though the essential role of vitamin D in modulating the immune response is well known, more evidence is needed on the effect of vitamin D in acute SARS-CoV-2 infection, and in long-COVID. Literature reports are controversial, and there is a need for more randomized controlled studies to better understand the protective role of vitamin D in the immune response to long-COVID [99].

### 7.5. COVID-19 and Hypothalamus–Pituitary Axis Disorders

COVID-19 has presented an important challenge to the medical community, and knowledge of how it impacts many biological systems is still limited. As we mentioned before, ACE-2 receptor expression has been found in gonadotroph, lactotroph, somatotroph, and corticotroph pituitary cells, thus supporting the idea of a direct COVID-19 pituitary infection [73].

Pituitary apoplexy is an endocrine emergency that requires immediate diagnosis and treatment. Recently, cases of pituitary apoplexy have been linked to COVID-19 infection [100] and could be the expression of its neurovascular phenotype, in which cerebrovascular endothelial damage can lead to progressive vascular syndrome or could be related to direct viral damage that enters the brain via the olfactory nerve or directly reaches the median eminence. In addition, as with SARS-CoV-1, SARS-CoV-2 may damage the hypothalamus–pituitary axis (HPA) via immune-mediated hypophysitis. COVID-19 may have indirect effects on the HPA through cytokines which cause functional hypopituitarism. Thyroid abnormalities of central functional hypothyroidism and hypoadrenalism have been reported in COVID-19 patients [101]. Studies on the hypothalamus-pituitary-adrenal axis involvement in post-COVID-1syndrome are still limited. Clinicians should be aware of possible endocrine dysfunction in patients with long-COVID complaining of unexplained fatigue, apathy, orthostatic dizziness, and loss of appetite, despite conventional treatment [75].

Because hypocortisolism and central hypothyroidism can last for years in SARS-CoV-1 survivors, it will be interesting to track the long-term pituitary hormonal profile in COVID-19 patients to see whether there is any pituitary-related morbidity [101].

Worse prognosis during and after the course of the disease has been described in patients with metabolic syndrome, including those with obesity, hypertension, and diabetes. Hypertriglyceridemia, hypertension, abdominal obesity, and decreased insulin resistance were significantly more prevalent in patients with hypopituitarism compared to the general population, due to altered body composition with modified lipid profile and increased proinflammatory cytokines and C-reactive protein (CRP) [102,103]. Obesity and altered body composition are also an important part of the clinical picture of other pituitary diseases such as Cushing’s disease, acromegaly, and polycystic ovary syndrome [104].

Obesity, along with altered glucose metabolism and immune system dysregulation, common features of the adult growth hormone (GH) deficiency syndrome, have also been found to be crucial determinants of the course of COVID-19 disease [105]. Cardiovascular risk is increased in these patients due to impaired fibrinolysis and elevated levels of proinflammatory cytokines such as TNF-α and IL-6, as mentioned above. Low-dose GH replacement therapy has improved these parameters, suggesting its involvement in inhibiting inflammatory syndrome and normalizing the fibrinolytic system [106]. Recent studies have shown that almost 30% of chronic heart failure patients had GH deficiency, with echocardiographic altered parameters, such as lower ejection fraction, higher end-systolic wall stress, lower performance of the right heart, and higher pressure in the pulmonary circulation, along with decreased ventilatory efficiency. GH deficient patients also showed higher all-cause mortality at follow-up [107].

### 7.6. COVID-19 and Gonads

The female reproductive axis is also vulnerable to COVID-19 infection, as ACE-2 receptors have been identified in the ovaries of premenopausal and postmenopausal women. Even though there are few data on the effects of COVID-19 infection on ovarian function, in a recent survey of patients with long-COVID, one-third of women reported menstrual irregularities or abnormal postmenopausal bleeding [76]. Estrogen deficit may also play a key role in the inflammatory process, with long-term cardiovascular consequences [108,109,110,111].

As recent data suggests, the IL-6-JAK-STAT3 axis is closely involved in the course of severe COVID-19 disease. High serum IL-6 has also been significantly correlated with mortality [108]. Estrogen facilitates the resolution of the inflammatory process through the regulation of the SOCS3 and STAT3 signaling pathways, leading to tissue remodeling and restoration of homeostatic conditions. Thus, estrogen deficiency is associated with increased serum concentrations of CRP, IL-6, and TNF-α [109,110]. Regarding the cardiovascular effects, estrogen improves the lipid profile by increasing HDL-cholesterol and lowering LDL-cholesterol and promotes blood clot formation, relaxation, and dilatation of blood vessels as well [108,111]. More studies are needed to clarify the long-term impact of SARS-CoV-2 on the female reproductive axis, beyond physical and psychological stressors.

On the other hand, some studies have shown that patients with severe COVID-19 had significantly lower testosterone levels compared to those with milder disease. Low levels of total testosterone and calculated free testosterone were found to be directly correlated with hyperinflammatory syndrome and associated with a longer course of the disease [112]. ACE-2 receptors have been identified in all testicular compartments, including Leydig, Sertoli, and germ cells. Recent evidence suggests that COVID-19 infection may affect not only testicular function but also spermatogenesis [76].

Low testosterone levels are also associated with a higher risk of metabolic syndrome components such as diabetes, dyslipidemia, hypertension, and cardiovascular events. Testosterone, and the more potent agonist of the androgen receptor (AR), dihydrotestosterone (DHT), bind to cytoplasmatic AR that is chaperoned by heat shock proteins. This will lead to the alteration of the myocardial and vascular cell behavior through the transactivation of a family of genes with androgen response elements [113].

Testosterone has also shown to have a direct rapid membrane effect on G protein-coupled receptors, leading to an increase in inositol triphosphate and diacylglycerol, with subsequent opening of calcium and potassium channels that induce relaxation of the coronary arteries [114]. Several meta-analyses and systematic reviews have clearly associated increased cardiovascular disease and mortality risk with lower testosterone levels [115]. Despite all the evidence indicating the vulnerability of testes to a COVID-19 infection, in addition to an acute reduction in testosterone concentration, recent studies have demonstrated that this fall in testosterone levels is reversible and resolves spontaneously after recovery from the disease [76].

### 7.7. COVID-19 and the Syndrome of Inappropriate Antidiuretic Hormone Secretion (SIADH)

Evidence suggests that patients with heart failure are more sensitive to low serum sodium levels than the general population; a mean serum sodium concentration of 138 mmol/L or less was a predictor of mortality due to reduced contractility in patients with mild to moderate heart failure [116]. The pathophysiological mechanisms which lead to hyponatremia in COVID-19 patients include the syndrome of inappropriate antidiuretic hormone secretion (SIADH), loss of sodium ions through the digestive system, a low salt diet, or the use of diuretics. Hyponatremia may also be considered a negative prognostic factor in patients diagnosed with COVID-19 [117].

Several hypotheses have been proposed to explain SIADH in pneumonia. In the setting of intravascular volume depletion, baroreceptors in the carotid sinus, carotid body, and aorta activate the renin-angiotensin system. This mechanism triggers baroreceptor-mediated, non-osmotic antidiuretic hormone (ADH) secretion [118].

Depression, anxiety or psychological stress, and pain associated with infections (such as COVID-19) stimulate the HPA, with ADH release. Stress also has a direct effect on the cortical neurons which stimulate the hypothalamus to secrete ADH. Another mechanism involves pneumonia-induced lung injury that results in a ventilation-perfusion mismatch which leads to hypoxic pulmonary vasoconstriction and inadequate venous return to the left atrium. In consequence, left atrial stretch is decreased, and ADH secretion increases. The high levels of inflammatory cytokines found in COVID-19 directly stimulate the non-osmotic release of ADH. Furthermore, these cytokines can injure the lung tissue and alveolar cells, leading to the hypoxic pulmonary vasoconstriction mentioned above, and SIADH [119,120].

The most common cardiac manifestations of hyponatremia are arrhythmias which frequently occur in patients with cardiovascular comorbidities, usually found in association with other electrolyte abnormalities. There are several case reports in the literature of severe hyponatremia associated with various types of conduction abnormalities in COVID-19 patients [121,122].

## 8. Conclusions

Long-COVID syndrome, along with its complications, MIS-A and MIS-C, represents an important challenge in the medical community. Underlying comorbidities can expose both COVID-19 adult and pediatric patients to a higher risk of negative outcomes not only during, but in the aftermath of the SARS-CoV-2 infection as well. These aspects can generate a significant impact on the health and quality of life of a long-COVID patient, as well as an economic burden.

Almost all endocrine disorders lead to cardiac dysfunction, from acute or chronic coronary syndrome to secondary complicated arterial hypertension, cardiac rhythm disorders, and, finally, acute heart failure. Consequently, the endocrine pathology is supposed to create, by its own natural disease history, an additive risk factor for MIS-A and this is the novel direction that we propose for future research.

Based on the information found in the literature, the time interval between the two pathologies, the reported symptoms, the laboratory evidence, and the administered therapy, we consider that there is an interdependent relationship between COVID-19 and MIS-A/C infection, respectively. Regarding long-COVID syndrome, we cannot say that there is an association between it and MIS-C. Of course, literature data related to the long-COVID syndrome are still scarce and potentially confounding. Diagnostic criteria from MIS-A/C have not been validated and differ globally, therefore, patients with milder MIS-A/C may not have been admitted or recognized.

## Figures and Tables

**Table 1:** Diagnostic criteria in MIS-C.

Diagnostic Criteria	Royal College Criteria	CDC Criteria	WHO Criteria
**Age**	Not stated	˂21 years old	0–19 years old
**Fever**	Persistent fever (≥38.5 °C)	Documented fever ≥ 38 °C or history of continuous fever ≥ 24 h	Fever ≥ 3 days
**Clinical changes**	Both criteria required: i. One or more organs affected ii. Additional criteria	Both criteria required: Severe disease with hospitalization At least two organs/systems affected: - Respiratory - Cardiovascular - Renal - Neurologic - Gastrointestinal - Dermatologic - Hematologic	At least two criteria: Rash, bilateral non-exudative conjunctivitis, or mucocutaneous inflammation (oral cavity, palms, and soles) Hypotension or shock Myocardial dysfunction, pericarditis, inflammation of the valvular system, or coronary anomalies Coagulopathy (increased PT or APTT or D-dimers) Gastrointestinal manifestations (abdominal pain, diarrhea, and vomiting)
**Inflammation markers**	All of the following criteria must be present: Neutrophilia Increased C-reactive protein Lymphopenia	Modification of one or more laboratory tests meaning inflammation (but not limited to) - Increased CRP - Increased ESR - Increased fibrinogen - Increased procalcitonin - Increased D-Dimers - Increased ferritin - Increased LDH - Increased IL-6 - Neutrophilia - Lymphopenia - Hypoalbuminemia	The presence of markers of inflammation, for example: - Increased ESR - Increased PCR - Increased procalcitonin
**Epidemiological link with SARS-CoV-2 infection**	Positive or negative RT-PCR	Current or recent infection sustained by any of the following: Positive RT-PCR Positive rapid antigenic test Positive serology Direct contact with a confirmed case in the last 4 weeks	SARS-CoV-2 infection sustained by any of the following: Positive RT-PCR Positive rapid antigenic test Positive serology Direct contact with a confirmed case

## Data Availability

Not applicable.
